# Continued Fever

**Published:** 1873-11-01

**Authors:** N. S. Davis


### Hospital Reports

### CONTINUED FEVER.

CLINICAL CASES IN THE MEDICAL WARDS OF THE MERCY HOSPITAL—SERVICE OF PROF. N. S. DAVIS, OCT. 17, 1873.

CASE I.—Mr. S——, a native of Norway, aged 25 years, was admitted into the Hospital two weeks since. He had been sick, about ten days previous to admission.

At that time his expression of countenance was dull; face suffused with dark redness; pro-labia red, dry, and the edges covered with sordes; the skin dry, harsh, and at a temperature of 104° F.; pulse 110, soft, weak; capillary circulation in surface slow; tongue covered with a thick, brown coat, dry in the middle; bronchial surfaces dry, and respiration unsteady; bowels distended, tympanitic, with from three to six thin, brownish discharges in the twenty-four hours; mind dull and wandering; a few small red spots over the abdomen and chest, with a large red spot, indicating the commencement of a bed sore, over the upper part of the sacrum. These symptoms indicated the existence of a severe grade of typhoid fever, in the second week of its progress. The soft, weak pulse; irregular respiration; and somnolency, indicated a dangerous degree of depression of the nervous centers, with, perhaps, a tendency to softening of the muscular structure of the heart; while the abdominal distension and intestinal evacuations equally pointed to the progress of serious lesions in the aggregated glands of the ilium. Hence, to sustain the nervous sensibility and muscular tonicity, on the one hand, and check the morbid action in the intestinal and mesenteric glands, on the other, constituted the two leading therapeutic indications. To fulfill the first, the patient was directed to have one teaspoonful of the following prescription, in a little sweetened water, every four hours:

℞.—Strychnia, gr. i.

Nitric acid, ʒ i.

Tinct. opium, ʒ ij.

Simple syrup, ℥ ss.

Water, ℥ iij ss.

Mix.

To fulfill the second, he was likewise directed to have one teaspoonfull of the the following emulsion, between each of the doses of the strychnia solution:

℞.—Oil turpentine, ʒ iij.

Oil wintergreen, gtts. xxx.

Tinct. opium, ʒ iv.

Pulv. g. arabic and sugar, aa. ʒ vi.

Rub together, and add water ℥ iv.

Mix.

The surface was to be sponged over with warm water twice a day; the discharges promptly removed from the ward; milk-whey given for drink, and thin wheat-flour and milk-porridge, in small quantities, at short intervals, for nourishment. The red spot over the sacrum was to be covered with cotton, moistened with carbolated oil. Under this management, the intestinal discharges became less frequent each day, until they occurred only once in from twenty-four to forty-eight hours, and the abdominal tympanitis nearly disappeared. The coat also came off the tongue, leaving it red and granular, with a dry strip through the middle. But the pulse continued soft and quick, the mind somnolent and muttering, with some subsultus. The emulsion was now omitted; the strychnia solution given once in six hours, and five grains of carbonate of ammonia, dissolved in a teaspoonful of camphor water, and camphorated tincture of opium, equal parts, given every two hours. The same nourishment and drink continued as before.

Four or five days have elapsed since these changes were made, and it is now between three and four weeks since the patient first took his bed. His skin is now soft, moist, and about the natural temperature; the flush is gone from the face; the lips and teeth are free from sordes; the pulse, though weak, is not more than 85 per minute; the tongue is clean and moist, but redder than natural; and the mind, though dull, sleeps more natural; in a word, the patient presents all the appearances of commencing convalescense. Still, the patient is very feeble, and will require the continuance of the same remedies, only at longer intervals, until the convalescence is well established.

Case II.—Mr. C——, aged 30 years, native of Ireland. He was admitted into the Hospital ten days since, near the end of the first week after his confinement to the bed. He presented the same general symptoms as the first case, at the time of admission. The abdomen was even more tympanitic, and the intestinal discharges quite frequent; but there was less somnolency, and decidedly more dryness and congestion of the mucous membrane of the bronchial tubes, accompanied by a mixture of dry and moist rhonchi over both sides of the chest, and some cough. There was, consequently, in this case, less danger from impairment of the nervous functions, or failure of the muscular force of the heart, but decidedly more danger from capillary congestion and passive exudation into the pulmonary structures, and serious lesions in the intestines. Indeed, the oppressed breathing, and tense abdominal distension, gave the case an aspect of extreme danger. To lessen the frequency of the intestinal discharges, and the tympanitic distension, he was given a teaspoonful of the turpentine and laudanum emulsion, as directed in the formulæ already stated, every four hours; but to relieve the congested condition of the mucous membrane of the bronchial tubes, he was given a teaspoonful of the following solution, between each of the doses of the emulsion:

℞.—Hydrochlorate of ammonia, ʒ iij.
Tart. ant. and pot., gr. ij.
Sulph. morph., gr. iij.
Syrup liquorice, ℥ iv.
Mix.

The hygienic regulations were the same as in the preceding case. In three or four days the intestinal discharges were limited to about one in the twenty-four hours, and the bronchial rhonchi were diminished; but the tympanitic distension of the abdomen remained unabated, and the pulse became more weak and frequent. The emulsion was discontinued, and the strychnia and nitric acid solution given every six hours; and instead of the hydrochlorate of ammonia mixture, he was directed to have the carbonate of ammonia, camphor and paregoric mixture, every two hours. On these remedies, with careful attention to nourishment, he has continued until the present time. Now we find him with the fever flush gone from his face; his skin slightly moist; temperature nearly natural; pulse soft and weak, but only about 100 per minute; only very slight rhonchi in the chest, but some dullness over the lower and posterior parts; tongue moist, and nearly clean, but red and fissured through the middle; mind clear, and little or no subsultus; and yet the abdomen was largely distended and very tense. The persistence, and even increase of this condition of the abdomen, after almost all the other phenomena indicated the commencement of convalescence, was pointed out as an unfavorable indication. It showed great want of contractibility, or actual paralysis of the muscular coat of the intestines, with extensive ulceration of the aggregated glands of the ilium. Allusion was made to some cases in which, the attending physician finding the tongue to become clean and moist, the skin cool, the pulse slower, and the mind clear, pronounced the patient convalescent, notwithstanding the continued distension of the abdomen; and yet they terminated fatally in a few days, either from a sudden return of copious evacuations, more or less mixed with blood, or from perforation of the intestines. In the present case, the strychnia and acid solution was continued, but the carbonate of ammonia and camphor was omitted, and the turpentine emulsion given in its place, with the hope of improving the condition of the bowels. It was stated that in similar conditions of the abdominal viscera, in the advanced stage of typhoid fever, nitrate of silver, with small doses of opium, had produced very favorable results. Also, that similar good results had sometimes followed the use of oxide of zinc, in two to three grain doses, combined with from half a grain to one grain of opium.

The clinic was closed by directing the attention of the class to a third case of typhoid, in the person of a young laboring man who had been admitted into the Hospital the day previous. The case was mild, presenting no serious complications.

The only treatment recommended was careful attention to sanitary regulations, bland nourishment, and six drops of hydrochloric acid every four hours, in sweetened water.

---

Every physician in regular practice, in city and country, should not only take one or more medical journals, but contribute as well. A large and lucrative practice, a high and influential position, are not alone sufficient to perpetuate a worthy name and reputation. These are perishable and will die out, when well-timed and well-recorded facts will last and establish true and genuine worth. Zimmermann remarked "that the greatest medical writers of any age were the best physicians." Those who communicate their views should rather be encouraged than decried.

